# Neutrophil-to-lymphocyte ratio and scoring system for predicting coronary artery lesions of Kawasaki disease

**DOI:** 10.1186/s12887-020-02285-5

**Published:** 2020-08-24

**Authors:** Ling-Sai Chang, Yi-Ju Lin, Jia-Huei Yan, Mindy Ming-Huey Guo, Mao-Hung Lo, Ho-Chang Kuo

**Affiliations:** 1 Department of Pediatrics and Kawasaki Disease Center, Kaohsiung Chang Gung Memorial Hospital, Chang Gung University College of Medicine, #123 Da-Pei Road, Niaosong District, 83301 Kaohsiung, Taiwan; 2 Department of Pediatrics, Chiayi Chang Gung Memorial Hospital, Chiayi City, Taiwan

**Keywords:** CRP, Coronary Artery Lesions, IVIG resistance, Kawasaki Disease, Neutrophil-to-Lymphocyte Ratio, Scoring System

## Abstract

**Background:**

Kawasaki disease (KD) causes coronary artery lesions (CAL) and is the leading cause of acquired heart disease in children. The aim of this study is to evaluate the risk factors and set-up a scoring system for predicting CAL of KD.

**Methods:**

We retrospectively reviewed a total of 478 patients diagnosed with KD. We compared age, gender, laboratory data, and treatment response in two groups and developed a scoring system for predicting CAL.

**Results:**

During the study period, 365 of these patients had complete medical records of coronary surveys by echocardiography. Anemia, hypoalbuminemia, C reactive protein (CRP), alanine aminotransferase, neutrophil count, and neutrophil/lymphocyte ratio (NLR) showed significant differences with CAL formation. We determined the cut-off value using a receiver-operating-characteristic (ROC) curve, and following multivariate logistic regression analysis, four independent risk factors demonstrated a significant difference with CAL formation, including CRP > 103 mg/L, NLR > 3.5, male gender, and intravenous immunoglobulin (IVIG) resistance. We established a score system based on the above evaluation, for which a ROC curve was performed, and a total score of ≥ 2 points showed a sensitivity of 60.8% and a specificity of 70.6%, with an area under the ROC curve of 0.696.

**Conclusions:**

Identifying children at risk is important in order to prevent CAL from developing. Four independent risk factors that can predict CAL formation were CRP > 103 mg/L, NLR > 3.5, male gender, and IVIG resistance. This first report incorporated NLR into score systems to predict CAL reinforces previously well-known risk factors for the CAL formation among KD patients.

## Background

Kawasaki disease (KD) is an acute febrile illness of unknown etiology characterized by systemic inflammation and vasculitis [1]. The disease most often affects infants between 9 and 11 months [2]. The most significant sequalae of KD is coronary artery lesions (CAL), such as coronary dilatation, aneurysms, narrowing, myocardial infarction, and valvular lesions [3, 4]. High-dose intravenous immunoglobulin (IVIG) (2 g/kg) is the standard treatment for KD, and its administration has been shown to significantly decrease the rate of CAL [5]. In patients that do not receive high-dose IVIG treatment, CAL occurrence was 25%, but patients who received high-dose IVIG had CAL occurrence of less than 5% [6].

Some individuals experience a period of coronary artery dilation after being diagnosed with KD and may experience changes in Z-score. However, new CALs are rarely detected by echocardiography at 6 weeks in KD children with normal measurements at baseline and 2 weeks after IVIG treatment [7]. In Skochko’s and Dominguez’s cohorts, 75–81% of patients who have an initial Z-score for the right coronary artery or left anterior descending artery of 2.5 or higher go on to develop coronary artery aneurysm [8, 9]. Therefore, identifying populations at a high risk of CAL is crucial to initiating early intervention. Otherwise, a large aneurysm can develop and lead to mortality and morbidity. Levels of calprotectin are elevated in pediatric patients with giant coronary artery aneurysms one-year post-KD [10]. Improved diagnostic tools and the development of risk-specific anti-inflammatory treatments have enabled intervention research for people at ultra-high risk. Most KD patients with CAL receive very brief treatment due to safety concerns. However, anti-inflammatory medications have not yet been approved for KD children and infants, mainly because exposure to anti-inflammatory medication, such as anti TNF-α, cyclosporin, and methylprednisolone pulse therapy, has been associated with such side effects as infection, renal toxicity, and hypertension [11–13]. Meta-analyses revealed that the use of corticosteroids may decrease the incidence of CAL in KD [14, 15]. A randomized controlled study on etanercept treatment showed positive improvement in CAL in patients with baseline abnormalities [12]. Another randomized controlled trial reported that adding ciclosporin to high-dose IVIG and aspirin in patients with KD significantly reduced the incidence of CAL (*p* = 0.010) in KD patients at a higher risk for IVIG resistance [11].

KD is the leading cause of acquired heart disease in children, and many risk factors associated with coronary artery lesions have been reported, including male gender, younger age, older children, IVIG unresponsiveness, and hypoalbuminemia [6, 16–23]. Son et al. developed the risk scores for predicting coronary artery aneurysms in a north American population and validated the score including baseline Z score ≥ 2.0, age < 6 months, Asian race, and C-reactive protein (CRP) ≥ 13 mg/dL [24]. Dionne et al. recently showed that adjunctive corticosteroid therapy with IVIG may be protective against progression of coronary artery aneurysms based on Son’s score [25]. Tremoulet et al. developed a scoring system for predicting coronary artery aneurysms: illness days ≤ 4 days, 1 point; bands ≥ 20%, 2 points; γ-glutamyl transpeptidase ≥ 60 U/L, 1 point; age-adjusted hemoglobin concentrations ≤ − 2, 1 point [26]. Sensitivity was 72.2%, specificity was 57.6%, positive predictive value (PPV) was 8%, and negative predictive value (NPV) was 97.5% at a total score of 2. Hua et al. reported a scoring system that included the following: male, 1 point; fever duration ≥ 8 days, IVIG resistance, 1 point; albumin ≤ 35.9 g/L, 1 point; monocyte ≥ 5.9%, 1 point [17]. The sensitivity and specificity were 51.4% and 68.2%, respectively, at a cut-off of 3. Many prediction models for IVIG resistance of KD have been developed, but a practical score with high sensitivity and specificity for predicting the risk of CAL in KD patients is still needed. Our aim in this study is to evaluate the risk factors of CAL and set up a scoring system for predicting CAL in KD patients.

## Methods

This study included a total of 478 patients diagnosed with Kawasaki disease at Kaohsiung Chang Gung Memorial Hospital from 2007 to 2018. Of those, 365 patients had complete medical records of coronary artery surveys by echocardiography. KD was diagnosed according to the criteria in the revised diagnostic guidelines for Kawasaki disease (the 5th revised edition) [27]. IVIG resistance was defined based on the published American Heart Association guidelines: “fever persistence (≥ 38 °C) or recurrence greater than 36 hours and up to 7 days following completion of the IVIG infusion.”

Of the 365 patients, 40 (11.0%) were treated with IVIG twice, and 4 patients (1.1%) were treated with IVIG three times. During the acute phase, all patients were treated with high-dose IVIG (2 g/kg) [28]. Laboratory data including complete blood count with differential count, aspartate aminotransferase (AST), alanine aminotransferase (ALT), CRP, albumin, and sodium were investigated, and data were obtained prior to IVIG treatment.

Echocardiography was performed in acute stage, and CAL were defined according to the Japanese Ministry of Health criteria: an internal lumen diameter greater than 3.0 mm in children of < 5 years of age or greater than 4.0 mm in children of ≥ 5 years of age or an internal diameter at least 1.5 times larger than the diameter of the adjacent segment, or if the morphology of the coronary lumen was obviously irregular, or a z-score ≥ 2.5 [3, 29]. We classified the patients into two groups: patients with CAL and patients without CAL [30]. We compared age, gender, laboratory data, and treatment response between the two groups.

Characteristics of patients, clinical features, laboratory data, CAL formation, and IVIG resistance were analyzed with IBM Statistical Product and Service Solutions Statistics 14 (New York, United States) using the Mann-Whitney test and chi-squared test. P < 0.05 was considered statistically significant. A receiver-operating-characteristics (ROC) curve was used to define the cut-off of each independent risk factor. Those factors were then analyzed again using binary logistic regression. Factors that showed a significant difference between the two groups were chosen to set-up a scoring system. The sensitivity, specificity, area under the curve of ROC (AUC), and PPV were evaluated through cross analysis.

## Results

We enrolled a total of 365 patients with complete medical records in this study. Tables 1 and 2 present the univariate analysis of the factors associated with CAL. Male gender (72.4% vs. 53.8%, p = 0.001) had a significantly higher percentage in patient with the CAL group than the without CAL group (Table 1). IVIG resistance was observed in 22.8% (29/127) of the CAL group and in 6.3% (15/238) of the no-CAL group during the acute phase (*p* < 0.001) (Table 1). We observed no statistically significant difference in the clinical features between the two groups (Table 1).

**Table 1:** Clinical characteristics of Kawasaki disease

	*Patient without CAL* *(n = 238)*	*Patient with CAL (n = 127)*	*p value*
***Age (year)***	1.5(0.8–2.6)	1.4(0.7–2.4)	*p* = 0.316
***Gender (Male)***	128(53.8%)	92(72.4%)	*p *= 0.001*
***IVIG resistance (+)***	15(6.3%)	29(22.8%)	*p* < 0.001*
***Clinical features***			
*** Oral changes***	230(96.6%)	125(98.4%)	*p* = 0.504
*** Conjunctival injection***	230(96.6%)	124(97.6%)	*p* = 0.754
*** Extremity changes***	233(97.9%)	125(98.4%)	*p* = 1.000
*** Rash***	230(96.6%)	122(96.1%)	*p* = 0.773
*** Lymphadenopathy***	28(11.8%)	15(11.8%)	*p *= 1.000
*** BCG injection***	130(54.6%)	58(45.7%)	*p* = 0.124

Data are expressed as median (range) or number (percentage). *BCG *Bacille Calmette-Guerin, *CAL *Coronary artery lesions, *IVIG *Intravenous immunoglobulin; *p < 0.05

No significant difference regarding CAL was found between different ages with cut-offs of 6 months old, 1 year old, 2 years old, and 3 years old (all p > 0.05) (Table 3). However, a higher incidence of CAL was found in younger children (6 months old vs. 1 year old vs. 2 year old: 41.7% vs. 37.5% vs. 36.3%), but this difference did not reach significance (p > 0.05).

**Table 2:** Laboratory data in patients with Kawasaki disease

	***Patient without CAL(n=238)***	***Patient with CAL (n=127)***	***p value***
***WBC× 10***^***3***^***/mm***^***3***^	13.07 ± 0.29	14.14 ± 0.48	*p*=0.183
***Hemoglobing/dl***	11.26 ± 0.07	10.96 ± 0.12	*p*=0.029*
***Platelets× 10***^3^***/mm***^***3***^	331.84 ± 7.29	343.88 ± 11.11	*p*=0.248
***Monocyte(%)***	6.24 ± 0.23	5.64 ± 0.24	*p*=0.359
***Eosinophil(%)***	3.17 ± 0.21	3.54 ± 0.34	*p*=0.631
***Basophil(%)***	0.72 ± 0.14	1.07 ± 0.20	*p*=0.061
***AST*** ***U/L***	67.95 ± 6.06	79.16 ± 10.23	*p*=0.40
***ALT*** ***U/L***	74.52 ± 7.06	92.93 ± 10.14	*p*=0.049*
***CRP*** ***mg/L***	79.31 ± 4.44	104.21 ± 7.29	*p*=0.006*
***Albuming/dL***	3.80 ± 0.03	3.59 ± 0.06	*p*=0.005*
***Sodium*** ***mEq/L***	136.35 ± 0.27	135.27 ± 0.50	*p*=0.072
***PLR***	59.39 ± 4.61	47.37 ± 2.61	*p*=0.124
***NLR***	2.73 ± 0.15	3.95 ± 0.39	*p*=0.004*
***Neutrophils× 10***^***3***^/mm^***3***^	7.79 ± 0.26	9.10 ± 0.40	*p*=0.019*
***Lymphocytes× 10***^***3***^***/mm***^***3***^	3.79 ± 0.11	3.52 ± 0.18	*p*=0.069

Data are expressed as mean ± standard errors. *ALT *Alanine aminotransferase, *AST *Aspartate aminotransferase, *CAL *Coronary artery lesions, *CRP *C-reactive protein, *NLR *Neutrophil/lymphocyte ratio, *PLR *Platelet/ lymphocyte ratio, *WBC *White blood cells; *p < 0.05.

Hemoglobin values were significantly lower in the CAL group (hemoglobin 10.96 ± 0.12 vs. 11.26 ± 0.07 g/dl, p = 0.029) (Table 2). The CAL group showed lower serum albumin (3.59 ± 0.06 vs. 3.80 ± 0.03 g/dL, *p* = 0.005). In contrast, CRP (104.21 ± 7.29 vs. 79.31 ± 4.44, *p* = 0.006), neutrophil count (9.10 ± 0.40 vs. 7.79 ± 0.26/mm^3^, *p* = 0.019), neutrophil/lymphocyte ratio (NLR) (3.95 ± 0.39 vs. 2.73 ± 0.15, *p* = 0.004), and ALT ( 92.93 ± 10.14 vs. 74.52 ± 7.06 U/L, *p* = 0.049) were significantly higher in the CAL group in the univariate analysis (Table 2). Both groups had similar leukocytes, platelets, percentage of monocytes, basophils, eosinophils, and lymphocytes, AST, sodium, and platelet/lymphocyte ratio (PLR) during the acute phase.

The Youden index and ROC analysis were applied to determine the cut-off value of CRP levels and NLR as potential predictors for CAL by plotting the proportion of true-positive results (sensitivity) vs. the proportion of false-positive results (1 - specificity). To predict CAL, multivariant logistic regression statistics identified four independent risk factors (CRP > 103 mg/L, NLR > 3.5, male gender, and IVIG resistance) based on six variables: CRP > 103 mg/L, ALT > 26 U/L, albumin > 3.5 g/dL, NLR > 3.5, male gender, and IVIG resistance (Table 4). We established a scoring model with four variables: CRP > 103 mg/L (multivariant p = 0.004), NLR > 3.5 (p = 0.035), male gender (p = 0.002), and IVIG resistance (p = 0.006) using the Youden index with a significant difference between patients with CAL and without CAL based on the above evaluation (Table 4). A score system was set up using the ROC curve. The total risk score was calculated as the sum of the individual scores, and the maximum total score for this risk model was 5.

**Table 3:** Cumulative distribution stratified by age

***Age***	***Patient without CAL(n=238)***	***Patient with CAL (n=127)***	***p value***
***6 month-old***	24(10.1%)	15(11.8%)	*p*=0.599
***1 year-old***	80(33.6%)	48(37.8%)	*p*=0.490
***1.5 year-old***	122(51.3%)	69(54.3%)	*p*=0.584
***2 year-old***	161(67.6%)	91(71.7%)	*p*=0.477
***2.5 year-old***	173(72.7%)	101(79.5%)	*p*=0.164
***3 year-old***	194(81.5%)	110(86.6%)	*p*=0.241

Data are expressed as numbers (percentage). *CAL* Coronary artery lesions

Table 5 shows the sensitivity, specificity, PPV, and NPV at each score cut-off. For the risk of CAL, we looked at all cut-off values and used ROC curve to determine the optimal cut-off of a total score of ≥ 2 points. The sensitivity for this cut-off score was 60.8%, and the specificity was 70.6% (see Table 5). The analysis resulted in an AUC of 0.696. Higher score implied lower sensitivity and higher specificity. Compared with patients who had a risk score of 0 to 1, those whose risk score was ≥ 2 had a much higher risk of having CAL (odds ratio 3.77, 95% confidence interval 2.40–5.93). Patients were classified as high risk if their diagnostic score was ≥ 2; otherwise, they were classified as low risk. The occurrent rate of CAL in the high risk group was 52.74% in contrast with only 22.83% in the low risk group. In Table 2, the p value of albumin was less than 0.05, so we added albumin as a prediction value. Albumin greater than or equal to 3.5 g / dL was equal to one point. The sensitivity of the score predicting CAL was much increased to 72.38%, and specificity was 60.59% after adding albumin with the cut-off value of the score greater than or equal to two points.

**Table 4:** Logistic regression models for differentiating Kawasaki patients with coronary artery lesions from those without coronary artery complications

	***Multivariant p value***	***Odd ratio (95% CI)***	***Score point***
***Male Gender***	*p*=0.002	2.35 (1.36-4.06)	1
***IVIG resistance (+)***	*p*=0.006	3.12 (1.38-7.04)	2
***CRP>103mg/L***	*p*=0.004	2.25 (1.31-3.88)	1
***NLR>3.5***	*p*=0.035	1.85 (1.04-3.28)	1

*CI *Confidence interval, *CRP *C-reactive protein, *IVIG *Intravenous immunoglobulin, *NLR *Neutrophil/lymphocyte ratio

## Discussion

In this study, we demonstrated that male gender, IVIG resistance, anemia, hypoalbuminemia, elevated CRP levels, higher neutrophils count, higher NLR, and higher ALT levels were all risk factors in KD patients who developed CAL. After performing multi-variant logistic regression analysis, we found male gender, IVIG resistance, NLR > 3.5, and CRP > 103 mg/L to be independent risk factors for predicting CAL formation and then developed a score model. At the cut-off of 2 points, the sensitivity was 60.8%, and the specificity was 70.6% with an AUC of 0.696. No significant difference was observed regarding CAL formation between different ages and clinical features.

CALs are the most significant complication in KD. Prompt treatment with high-dose IVIG can lower the coronary artery aneurysms rate from 20–25% to 3–5%. However, 3–5% patients still develop CAL that need a lifetime of follow-up and treatment. Early classification of patients with high risks can alert the clinician to follow up frequently or to try different anti-inflammatory treatment options, such as steroids [31, 32].

Many researchers have developed score systems for the early detection of IVIG resistance by NLR, but few score systems are for predicting the subsequent CAL formation by NLR [33–35]. In infants younger than one year old, NLR with cut-off value of 2.51 is useful to predict the IVIG resistance [36].

Nakano et al. demonstrated that age at onset, CRP, and platelet count can all be used to predict patients at a high risk for coronary lesions [37]. Beiser et al. constructed a risk classification tool based on baseline hemoglobin level, neutrophil count, platelet count, and body temperature [38]. Kim et al. found that incomplete KD, IVIG resistance, longer fever duration, and the rs7604693 genetic variant in the *PELI1* gene were all risk factors for the formation of CAL [39].

Previous studies found that physical immune system responses to systemic inflammation included marked neutrophilia and lymphocytopenia [40]. Neutrophils indicate active non-specific inflammation, and lymphocytes represent the regulatory pathway of the immune system. Egami score or Kobayashi scores have pointed out the importance of neutrophil percentage for predicting non-responsiveness to IVIG [41]. The NLR represents the balance between inflammation and immune regulation. Amano S et al. have clearly demonstrated that systemic inflammation occurs in KD patients and mainly affects the cardiovascular system [42]. Therefore, NLR may help reflect systemic inflammation and immune system response in patients with KD. Kee-Soo Ha et al. demonstrated that NLR can predict coronary aneurysm development and IVIG resistance of KD [43]. Furthermore, Seiichiro Takeshita et al. revealed that the combination of NLR ≥ 3.83 and PLR ≥ 150 for predicting IVIG resistance in KD has both high sensitivity (72%) and specificity (67%) [44]. NLR can be a reliable predictor for IVIG resistance, which can be associated with an increased risk for CAL in children with KD [18]. In our study, NLR is an independent risk factor for CAL formation with the cut-off of NLR > 3.5, but PLR showed no significant difference between patients with CAL and those without CAL. Higher levels of NLR may represent higher inflammation levels and are associated with coronary damage.

This study had some limitations. First, all of our patients were from a single institution, and all of the KD patients were treated with the initial therapy of high-dose IVIG. Outcomes may differ from different initial therapies, such as IVIG alone or IVIG plus steroids. However, our laboratory data were all obtained prior to IVIG treatment, so different treatment options may not influence our scoring system. Second, only typical KD patients were included during patient selection, which may have involved some bias. The score might only be used in typical KD patients. Third, it was difficult to know whether these patients had previous cardiac lesions prior to the onset of KD. Another prospective study with more patients with KD will be helpful to confirm our results.

**Table 5:** Scoring system for predicting coronary artery lesions

*Cut point*	*AUC*	*Sensitivity*	*Specificity*	*PPV*	*NPV*
*1 point*	0.696	90.4%	27.2%	39.8%	84.2%
*2 points*		60.8%	70.6%	52.4%	77.2%
*3 points*		29.6%	90.6%	62.7%	70.8%
*4 points*		16.8%	97.0%	75.0%	68.7%

*AUC *Area under the curve of receiver-operating-characteristics, *NPV *Negative predictive value, *PPV *Positive predictive value

## Conclusions

In this study, we developed a scoring system to predict CAL in KD. Our score model included male gender, IVIG resistance, NLR > 3.5, and CRP > 103 mg/L. At the cut-off point of 2 points, the sensitivity was 60.8%, and the specificity was 70.6%, with an AUC of 0.696. This scoring system can help clinicians with early recognition of high-risk patients and can lead to different treatment options.

## Abbreviations

ALT: Alanine aminotransferase
AST: Aspartate aminotransferase
AUC: Area under the curve of ROC
BCG: Bacille Calmette-Guerin
CAL: Coronary artery lesions
CI: Confidence interval
CRP: C reactive protein
IVIG: Intravenous immunoglobulin
KD: Kawasaki disease
NLR: Neutrophil/lymphocyte ratio
NPV: Negative predictive value
PLR: Platelet/lymphocyte ratio
PPV: Positive predictive value
ROC: Receiver-operating-characteristics
WBC: White blood cells

## Availability of data and materials

The datasets used and/or analysed during the current study available from the corresponding author on reasonable request.
